# Barriers and facilitators to diabetic retinopathy screening within Australian primary care

**DOI:** 10.1186/s12875-021-01586-7

**Published:** 2021-11-30

**Authors:** Matthew J. G. Watson, Peter J. McCluskey, John R. Grigg, Yogesan Kanagasingam, Judith Daire, Mohamed Estai

**Affiliations:** 1 The Australian e-Health Research Centre, CSIRO, 147 Underwood Avenue, Floreat, WA 6014 Australia; 2 Save Sight Institute, The Faculty of Medicine and Health, The University of Sydney, Sydney, Australia; 3 School of Medicine, University of Notre Dame Australia, Fremantle, Australia; 4 St John of God Public and Private Hospitals, Midland, Australia; 5 School of Population Health, The Faculty of Health Sciences, Curtin University, Bentley, Australia; 6 School of Human Sciences, The University of Western Australia, Perth, Australia

**Keywords:** Diabetic retinopathy, Screening, Eye, Photography, Diabetes, Primary care

## Abstract

**Background:**

Despite recent incentives through Medicare (Australia’s universal health insurance scheme) to increase retinal screening rates in primary care, comprehensive diabetic retinopathy (DR) screening has not been reached in Australia. The current study aimed to identify key factors affecting the delivery of diabetic retinopathy (DR) screening in Australian general practices.

**Methods:**

A descriptive qualitative study involving in-depth interviews was carried out from November 2019 to March 2020. Using purposive snowballing sampling, 15 general practitioners (GPs) were recruited from urban and rural general practices in New South Wales and Western Australia. A semi-structured interview guide was used to collect data from participants. All interviews were conducted over the phone by one facilitator, and each interview lasted up to 45 min. The Socio-Ecological Model was used to inform the content of the interview topic guides and subsequent data analysis. Recorded data were transcribed verbatim, and thematic analysis was conducted to identify and classify recurrent themes.

**Results:**

Of 15 GPs interviewed, 13 were male doctors, and the mean age was 54.7 ± 15.5 years. Seven participants were practising in urban areas, while eight were practising in regional or remote areas. All participants had access to a direct ophthalmoscope, but none owned retinal cameras. None of the participants reported performing DR screening. Only three participants were aware of the Medicare Benefits Schedule (MBS) items 12,325 and 12,326 that allow GPs to bill for retinal screening. Seven themes, a combination of facilitators and barriers, emerged from interviews with the GPs. Despite the strong belief in their role in managing chronic diseases, barriers such as costs of retinal cameras, time constraints, lack of skills to make DR diagnosis, and unawareness of Medicare incentives for non-mydriatic retinal photography made it difficult to conduct DR screening in general practice. However, several enabling strategies to deliver DR screening within primary care include increasing GPs’ access to continuing professional development, subsidising the cost of retinal cameras, and the need for a champion ace to take the responsibility of retinal photography.

**Conclusion:**

This study identified essential areas at the system level that require addressing to promote the broader implementation of DR screening, in particular, a nationwide awareness campaign to maximise the use of MBS items, improve GPs’ competency, and subsidise costs of the retinal cameras for small and rural general practices.

**Supplementary Information:**

The online version contains supplementary material available at 10.1186/s12875-021-01586-7.

## Background

Diabetic retinopathy (DR) is one of the earliest clinically observable complications of diabetes mellitus, and it is a major cause of vision impairment and blindness [1, 2]. According to Australia’s Eye Health Survey 2016, among adults 40 years of age and older, the sampling weight-adjusted prevalence of any DR and vision-threatening diabetic retinopathy (VTDR) was 39.4% (95% CI, 33.1–46.1) and 9.5% (95% CI, 6.8–13.1), respectively [3]. The Blue Mountains Eye Study reported that the 5-year DR cumulative-incidence and DR progression were 22.2 and 25.9% in Australia [4]. Among the global population, approximately 93 million people were affected by DR, 17 million were affected by proliferative DR, 21 million were affected by diabetic macular oedema, and 28 million had VTDR [5]. Vision loss affects the national economy by losing productivity and earning capacity [6]. With projected increases in people with diabetes, the economic burden of sight-threatening complications of diabetes will invariably increase [7, 8].

Approximately half of the people with proliferative DR who do not obtain prompt treatment will be blind in 5 years [9]. Critically, detecting DR at an early stage, followed by timely and adequate treatment, can prevent more than 90% of diabetes-related vision loss [10]. The early stages of DR are asymptomatic, and therefore, systematic retinal screening is essential. The Australian National Health and Medical Research Council (NHMRC) recommends biennial retinal screening for people with diabetes but no evidence of DR and annual retinal screening for Indigenous people with diabetes and those known to have DR [11].

Retinal photography has moved retinal examination away from the traditional method of using slit-lamp biomicroscopy or ophthalmoscope with mydriatics performed by optometrists or ophthalmologists [12]. Non-mydriatic retinal photography has become a more popular DR screening technique, with a sensitivity of at least 80% compared with a reference standard [13]. This technique is easy and safe and can be carried out by nurses or other trained personnel. Retinal photography also enables tele-retinal screening, referring to teleophthalmology whereby retinal images can be transferred online to offsite eye experts for further opinion [14]. This approach can enhance patients’ access to eye care, particularly for those in remote regions. Tele-retinal screening for DR has been successfully implemented in other health programs like the UK, USA, and Singapore [15–18].

Medicare is Australia’s universal health insurance scheme equivalent to the UK National Health Service (NHS). In 2016, the Department of Health, Australia introduced two new Medicare Benefits Schedule items (MBS items 12,325 ‘for Aboriginal and Torres Strait Islander population’ and MBS items 12,326 ‘for general population’) to support retinal photography and image reporting by general practitioners (GPs) rather than ophthalmologists or optometrists [19]. Pre-introduction modelling suggested that over 400,000 patients with diabetes would be screened after introducing MBS items for retinal photography. However, data from the Department of Health show that these MBS items have been massively underused [19]. For instance, between July 2016 and July 2020, only 4256 Indigenous Australians and 3776 non-Indigenous Australians were screened by GPs with claims on the MBS [19].

Despite the appeal of retinal photography in facilitating retinal screening, including incentives through Medicare, comprehensive DR screening has not been achieved in Australia. Studies have shown that only 50–77% of non-Indigenous Australians and 20–44% of Indigenous Australians receive appropriate retinal screening [20–23]. This gap between the recommended NHMRC retinal examination guidelines and actual DR screening rates relates to several factors, including patients’ knowledge of both the condition and the need for retinal screening, doctor-patient’s communications, travel, operating costs, infrastructure, and time constraints within general practice [24–26].

There have been some qualitative analyses of the barriers to DR screening in the Australian general practice setting, but all were conducted before introducing MBS items for retinal photography in 2016 [27–31]. The current study aimed to explore key factors affecting the provision of DR screening for patients with diabetes from GPs’ perspectives and determine effective strategies to enhance the successful integration of DR screening into practice workflow.

## Methods

Qualitative research, utilising in-depth interviews with primary care providers in Australia, was conducted between November 2019 and March 2020. A descriptive qualitative content analysis approach, guided by the existing theoretical framework and naturalistic inquiry, was used to interpret meaning from data generated from the participants’ responses [32]. The current study was performed in accordance with the Standards for Reporting Qualitative Research [33] (supplementary file 1). The study was approved by the CSIRO Health and Medical Human Research Ethics Committee (Ref no: 2019_070_LR).

### Theoretical framework

**Fig. 1:**
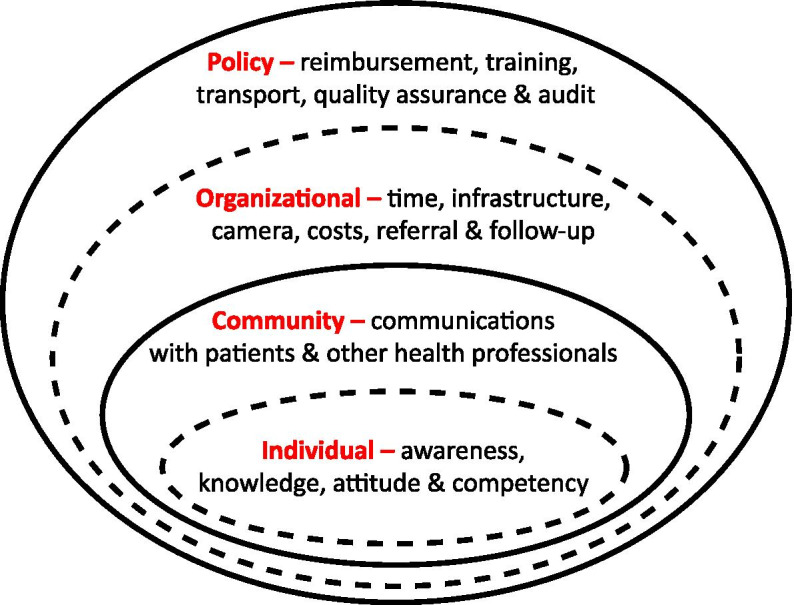
Socio-ecological model illustrating interactions of primary care providers with their environment at each level

The Socio-Ecological Model was used to inform the content of the interview topic guides and subsequent data analysis [34, 35]. This framework describes the complex interactions between various factors at the individual, community, organizational and policy levels, and their influence on the intended outcomes (Fig. 1). Also, the interview topic guides were informed by other studies [24–29, 36–39]. The questions explored GPs’ awareness of MBS items for retinal photography, competency, knowledge about their role in this service and barriers to performing DR screening for their patients (supplementary file 2).

### Participants

The purposive snowballing sampling technique was used in the recruitment, commonly used in qualitative research to ensure maximum inclusivity [40]. First, purposeful sampling was applied, and then more participants were recruited using snowball sampling. GPs registered in Australia, providing care to patients with diabetes, and access to the MBS item numbers 12,325 and 12,326, were invited to participate in this study. Invitation letters along with participant information consent forms were distributed through local GP networks (e.g., NSW rural doctor network). Out of 50 GPs invited through purposive snowballing sampling, only 15 Australian GPs from urban and rural practices in New South Wales (NSW) and Western Australia (WA) were enrolled. Those who agreed to join the study were then contacted via emails to arrange a suitable time for telephone interviews.

The prevailing concept for sample size in qualitative studies is data saturation - the point at which no additional or new information is identified in the qualitative data [41]. It has been reported that saturation often presents within the first 12 interviews, although essential components for metathemes can occur as early as six interviews [42]. Nevertheless, the recruitment of new participants continued until the COVID-19 related temporary shutdown in March 2020 interrupted further recruitment and data collection. However, it was judged by the authors that data saturation had been reached by that point.

### Data collection

Semi-structured interviews were the most applicable method for data collection to meet our research aims. All interviews were facilitated by the lead author (MW). Interviews were conducted via Webex™ videoconference system (CISCO) and were recorded digitally. Interviews were conducted in English, and each lasted up to 45 min. In addition, the lead author (MW) secured verbal consent and collected demographic information from each participant before the interview started.

### Data analysis

A deductive approach was used to analyse interview data guided by the theoretical framework (socio-ecological model) as a basis for coding and themes development [32, 43]. Recorded data from the interviews were transcribed verbatim by a professional transcription service. First, JD read all transcripts to familiarise herself with the whole set of data and created a categorisation matrix based on the existing theoretical framework. All the transcribed data were coded using the categorisation matrix, and themes (repeated patterns of the meaning) were generated based on the study’s aim [32, 43]. Then, JD, MW and ME reviewed the categories and themes independently and collectively. Finally, findings were discussed amongst the authors to reach a consensus on emergent data categories, subthemes and themes.

The credibility and dependability were maintained to ensure the rigour of the study. Dependability was maintained via audit trailing to record all the researchers’ notes throughout the study. Credibility was improved through an in-depth study of the data or transcripts by independent authors to ensure that emerging themes and subthemes were accurately represented the participants’ views.

## Results

Thirteen male and two female GPs aged 54.7 ± 15.5 years completed phone interviews. All interviewees were based in WA or NSW, and 60% were practising in urban regions. Four GPs had attended medical schools overseas (namely, one in the UK, two in South Africa and one in India), while the remaining 11 GPs had attended Australian medical schools. A summary of participants’ characteristics is presented in Table 1.

**Table 1:** Characteristics of participants (*n* = 15)

	Number of GPs
**Age in years**	54.7 ± 15.5 years Range 29–73 years
**Gender**	
Male	13
Female	2
**Location**	
WA	7
NSW	8
**Current practice settings**	
Metropolitan areas	9
Regional or remote areas	6
**Years of clinical experience**	
1–5 years	3
6–15 years	3
16–25 years	1
26–40 years	3
> 41 years	5

None of the interviewees conducted DR screening in general practice. All interviewees had access to a direct ophthalmoscope, but none had retinal cameras or image analysis software in their general practices. Only three interviewees were aware that MBS items 12,325 and 12,326 allowed GPs to bill for retinal screening using a non-mydriatic retinal camera.

Seven themes were identified as related to potential facilitators and barriers to more widespread implementation of DR screening within Australian primary care (Table 2).

**Table 2:** Barriers and facilitators to implementing DR screening in general practice

**Barriers**	
• GPs’ incompetency to make an accurate diagnosis of DR.	
• Costs of retinal cameras, particularly for small and rural practices.	
• Lack of time within the practice.	
• Unawareness of Medicare incentives for non-mydriatic retinal photography among Australian GPs.	
• Uncertainty about GPs’ role in the retinal screening program.	
**Facilitators**	
• Increase GPs’ access to training on DR grading and detection.	
• A nationwide awareness campaign to increase the uptake of MBS items and roll out DR screening in general practices.	
• Subsidise the cost of retinal cameras, particularly for small or rural general practices.	
• The need for a champion (e.g., nurses) within the practice to ensure the integration and continuity of the service.	
• Uncouple the need to perform both retinal photography and image reporting by GPs.	
• Take advantage of artificial intelligence to facilitate automated DR screening.	

### GPs awareness and impressions of MBS items 12,325 and 12,326

Although MBS items for DR screening have been introduced since 2017, only three interviewees were aware of this. After being notified through their study participation, some of the GPs found these MBS items to be a positive development….. “If it [retinal photography] can be billed in addition to a time-based item number, it can provide the financial incentive” (GP 12). Participants indicated that introduction of MBS items for retinal photography would encourage patients to attend screening appointments……. “this would give patients more choice and feel more comfortable coming to the GP to have eye screening” (GP 14).

The requirements of the MBS item descriptors both to perform retinal photography and interpret the retinal images were perceived as a significant barrier to conducting DR screening by GPs. In addition, participants experienced difficulties with keeping up with new skills, and therefore uncoupling these two components was suggested, whereby GPs could capture retinal images while letting eye care experts review and interpret the retinal images at a later time ..…. “it’s impossible to keep up with every skill but might be easier to take an image and flick it to an alternative source” (GP 15).

### GPs’ role in DR screening

Participants had a strong belief in their role in the screening and management of chronic diseases. They considered themselves as ‘gatekeepers’ of the healthcare system.…. “We’re at the front line, and our role is critical in identifying any complications of chronic disease” (GP 3).

Almost all interviewees indicated that they were interested in DR screening but that there are several barriers. Some interviewees felt that DR screening is not appropriately done compared to other countries…. “In the UK, when patients diagnosed with diabetes, they put on a national registry and every two years invited to attend a screening” (GP 13). Only one participant had previously done some DR screening but discontinued the service because the quality of images was poor and difficult to interpret. Although they recognised DR screening benefits, interviewees felt they had a limited role in the current DR screening program. They held the view that optometrists or ophthalmologists were more appropriate and equipped for retinal screening roles than GPs.…… “I always do encourage them [my patients] to go see optometrists” (GP 3). In addition, the interviewees considered themselves the first step in the referral pathway and their roles not extending beyond that…… “currently I see our role often as a referral source to either optometrists or ophthalmologists” (GP 12).

In regions with a high incidence of diabetes and poor engagement with primary care, getting people for eye screening is even more challenging. Participants identified that patients’ compliance could influence the adherence to DR screening guidelines. They suggested that providing comprehensive care to patients with diabetes could improve DR screening rates and may avoid patients from visiting multiple clinicians…… “We do have a lot of patients, and it would be ideal for us just to do all of their diabetic care plans in one appointment” (GP 1).

### GPs’ competency in DR detection and grading

Although training provided during medical school was insufficient, some participants believed that retinal screening is not beyond the scope of well-trained GPs. While others felt it is too hard to conduct DR screening, and they are incompetent even at doing fundoscopy…… “It’s certainly something we learn about in medical school, but the training was quite minimal” (GP 1). However, participants indicated that upskilling GPs to provide eye screening could help to ensure the continuity of diabetic care, patients’ attendance, and the ability to screen those [in rural areas] with limited access to optometrist or ophthalmologists…… “lots of advantages of having a GP upskilled and able to provide retinal screening” (GP 11).

Except for two, all interviewees were concerned about lacking the skills and competence to detect and grade DR on retinal images accurately. … ….. “I don’t think we [GPs] would feel confident to make that diagnosis” (GP 1).

### Costs

Except one, all GPs commented that the existing incentives from Medicare do not justify the cost of retinal cameras and ongoing expenses including training and time spent on DR screening……. “That $50 [rebate rate] is a pretty measly amount” (GP 8)……. “just like spirometry, we only get $35 to do that, which is a fairly time-consuming” (GP 1). The cost of the camera is commercially not viable, given that the number of patients seeking eye screening remains low. Participants felt that the capital cost is a major barrier even if GPs had the knowledge and skills to acquire and interpret the images quickly.…… “The returns on investment certainly are a big issue, especially if it [camera] just languishes on a shelf somewhere” (GP 8).

Solo GPs, small practices or rural practices were particularly identified as needing a further governmental subsidy to help with the costs of retinal cameras to perform DR screening services for their patients……. “We would struggle to buy retinal camera…… the government should aid funding the equipment and training” (GP 14). In addition, participants raised concerns about continuous changes in Medicare policy, which may affect GPs’ decision to purchase a retinal camera or invest time in training …… “MBS is changing all the time, so you might buy a camera only to find that the government no longer would reimburse this” (GP 8).

### Time constraints

Ten interviewees cited that the time needed for retinal photography, interpretation of images and learning new skills was cited as a barrier to performing DR screening.…… “Time limitation is GP’s greatest enemy along with the cost of technology” (GP 13).

Participants also had a concern about time devoted to training and operating the retinal camera within the practice. Some GPs see their patients twice a year, providing clinical assessments, advice on diets, adjusting medications, discussing side effects, and managing blood pressure. Therefore, doing retinal photography by GPs during that appointment may add further burdens……. “It’s a time factor again in learning a new skill to operate another machine, training staff, and use it” (GP 3). In addition, as the incidence of diabetes increases, performing eye screening for patients with diabetes would be more challenging in the coming years…… “The target population [patients with diabetes] is so large” (GP 2).

### The need for a champion ace to take the responsibility of retinal screening

Eleven interviewees indicated that for DR screening to become a part of an ongoing practice workflow, it must be championed by dedicated staff within the general practice……. “even if the machine were free, you would still have to get someone to take it on” (GP 1). Many GPs have a nurse practitioner who develops a diabetes care plan for patients, performs routine tests, and follow-ups. Nurses or diabetes educators were suggested to play a major role in facilitating DR screening by taking the responsibility of retinal photography.…… “Since most of these practices [large clinics] have a nurse doing the preliminary care plan, the images could be taken before the patient even seen by GPs” (GP 15). In addition, nurses could help with the practice management, including scheduling appointments for eligible patients, recalls and referrals to specialists…… “They [nurses] on our backs to arrange the necessary referrals and the paperwork” (GP 9).

### The role of artificial intelligence (AI) in facilitating DR detection

Almost all interviewees were enthusiastic about the use of AI and its potentials to improve DR detection and sav GPs’ time without the need for eye experts’ opinions. Such technology can be used to save thousands of patients’ vision, prevent significant deterioration of vision and inevitably free-up huge costs.…… “if an algorithm [AI] could tell me whether there is diabetic retinopathy or not, I could refer them straight to an ophthalmologist” (GP 8)…... “it [AI] can be better than many specialists in picking up these” GP3.

GPs indicated that the use of AI could help to educate patients on the spot about diabetes and its sequences on patients’ visions and encourage patients for better control of blood glucose.…. “it would be a great opportunity to educate patients …. which could motivate them to control their blood glucose level better” (GP 8). In addition, the use of AI in DR screening would have positive impacts on patient’s acceptance of results……. “patients would be quite accepting of the AI results, almost more so than my clinical diagnosis” (GP 12).

Some GPs flagged that the use of AI in clinical practice may raise new problems like costs of upgrading technologies and medico-legal issues associated with the accuracy of the screening results.…. “inevitably, it [AI] needs to be updated and upgraded” (GP 12)….. “If GPs are expected to override the judgment of the AI then that could be an issue” (GP 1).

## Discussion

Although all vision loss due to DR is preventable with early detection and timely treatment, yet DR remains one of the common causes of vision impairment and blindness worldwide [1, 5]. The main findings of this study were that most interviewees were unaware of MBS items for retinal screening, did not carry out retinal screening in practice and felt that their primary role was to refer patients requiring DR screening to optometrists or ophthalmologists. These concerns were mainly attributed to the lack of skills to diagnose DR accurately, costs of retinal cameras, time constraints within the practice and the absence of dedicated staff to take the responsibility of retinal photography.

The number of times GPs screened for DR between 2016 and 2020 was far lower than they initially predicted [19]. Most interviewees were unaware of the MBS items for retinal photography and DR screening requirements in general practice. This is coupled with GPs lacking competence in interpreting retinal images and uncertainty about their role in this service. These system-level factors seem to underpin the underuse of MBS items for retinal screening and willingness to refer any patient requiring retinal examination to optometrists. If the intent of MBS items is to be successfully implemented, then there is a need to invest more in a nationwide awareness campaign amongst GPs to encourage the use of these items. Previous studies suggested increased opportunities for continuing professional development (CPD) as a viable solution for lacking skills and confidence to diagnose DR accurately [27, 29, 44]. For instance, the University of Melbourne launched an online Self-Directed Diabetic Retinopathy Grading Course in 2017 to train GPs in images’ interpretation and make appropriate referral timelines for DR [45].

Costs of the retinal cameras (around $30,000 AUD) and the current incentive structure through Medicare were perceived as DR screening barriers in general practice. Such system-level barriers have been observed in previous studies [27, 38]. Governmental assistance, particularly for small or rural general practices, could positively impact the uptake of MBS items and facilitate DR screening into practice workflow. Given that most interviewees reported that they are not competent in diagnosing DR from retinal images, some suggested uncoupling the need for GPs to undertake both retinal photography and image reporting. Letting GPs concentrate on capturing retinal images for forwarding to eye care experts to review. This was also considered by the Medicare Services Advisory Committee when approving these MBS items in 2014 [46].

The interviewees reported insufficient time to conduct retinal photography as a primary barrier to DR screening in general practice. This provider-level barrier was consistent with previous studies indicating GPs’ concerns about the time required for training and the time involved in taking and reading images [27, 44]. In the current study, interviewees recognised the need for a champion and took responsibility for DR screening to become part of an ongoing practice workflow. The need for dedicated staff within general practices has been observed previously [27]. Nurses could play a fundamental role in organising appointments and collecting retinal images from patients when attending regular check-ups. The availability of the MBS items for general practice DR screening and the MBS item number 10997 for chronic disease management by practice nurses would encourage GPs to support the delivery of DR screening.

The recent emergence of Artificial Intelligence (AI), particularly deep learning, provides an accurate, objective and timely automated reading for DR from retinal images [47–49]. Interviewees’ attitudes to these technologies were overwhelmingly positive provided the sensitivity of the AI system is high enough, although some flagged there could be medico-legal issues concerning the responsibility for the results. The use of AI could help uncouple the two components of the MBS items for general practice DR screening. In addition, its use in clinical practice would significantly enhance the opportunity to increase DR screening rates and improve adherence to the NHMRC screening criteria.

GPs see patients with diabetes more than other health professionals and maintain good, long-standing relationships with many of their patients. Therefore, GPs are well-placed for conducting an opportunistic screening. Interviewees considered themselves gatekeepers of the healthcare system and were keen to play a more significant role in DR screening if their needs could be met. Therefore, it was reasonable to introduce the MBS items to encourage GPs for DR screening in general practice in targeting this important condition.

The present study fits in the international context as an existing theoretical framework [50, 51] was adopted to inform the interview topics guide and qualitative data analysis. Although findings from this study were specific to the Australian health system, several lessons can be drawn that can enrich the current literature. Our findings were consistent with the current literature [25, 26], which cited costs, infrastructure, and time constraints as main barriers to DR screening in primary care. The present study also identified other provider and system-level factors, including competency, training and reimbursements that may affect rolling out DR screening in primary care. Despite insurers’ support (e.g., incentives through Medicare) to implement a successful DR screening program within Australian primary care, this has been insufficient to increase the screening rates. The barriers within the same theme may interact with more than one of the healthcare system levels (i.e., system, provider and patient) [25]. For instance, unawareness of MBS items and lack of training were system-level barriers that caused health providers to underuse DR screening and uncertainty about their role in this service. Such shortcomings contributed to reduced uptake of DR screening and poor coordination and integration of retinal screening service, which require resolving the obstacles impacting all three levels, with the patient at the centre, to maximise DR screening rates and consequently reduce the risk of blindness from diabetes.

Against this background, there is a need to develop a better framework of crucial enablers that underpin the successful model of DR screening in general practice. Along with insurers’ support, the following enabling strategies can be adopted, which are consistent with other successful international DR screening models, the NHS retinal screening program [15]:

- Increase GPs’ access to training and CPD on DR grading and detection.
- Lunch nationwide awareness campaigns to increase the roll out of DR screening in primary care similar to other successful Australian screening programs (National bowel cancer screening program).
- Subsidise the cost of retinal cameras, particularly for small or rural general practices.
- A champion ace to ensure the integration and continuity of eye screening service within the general practices. e.g., delegating a nurse or health educator to organise appointments/follow-ups and acquire images.
- Uncouple the need to perform both retinal photography and reporting by GPs. e.g., letting GPs grade the images while nurses organise appointments and capture images.
- Establish a diabetes registry to identify eligible individuals for screening, recall system and effective referral pathways.
- Establish auditing and quality assurance schemes to be integrated into the service.

## Limitations of the study

The purposive sampling methods used may restrict the variation of the participating sample. The majority of participants were male doctors, meaning that the views and experiences of female doctors in Australia were not well represented in our data. Thus, in-depth interviews with more GPs from both genders may have enabled more detailed reflections on barriers. Despite this, the study sample included GPs with varying experiences and ages from different practice settings and locations. The present study explored critical factors affecting the delivery of DR screening from GPs’ perspective; thus, further research is needed to identify other barriers in the healthcare system or from the patients’ perspectives. In addition, it would be worth investigating other common themes that have not been covered in the present study [25, 26], in particular, patients’ education, staff communications, infrastructure, coordination and integration of the service, quality assurance and the need for diabetes registry.

## Conclusion

There has been an overestimation that introducing MBS items for retinal photography would offer adequate incentives for GPs to incorporate DR screening into practice workflow, yet significant barriers exist. The present study identified essential content themes at individual, provider and system levels that provide crucial and pragmatic additional insights into what needs to be addressed to promote widespread DR screening within primary care. Providing accessible one-stop comprehensive diabetes care service, reviewing the current incentive structures, subsidising the cost of retinal cameras, and improving GPs’ competency may all contribute to the success of DR screening in general practices and reduce preventable blindness.

## Supplementary Information

**Additional file 1.** Standards for Reporting Qualitative Research (SRQR).

**Additional file 2.** Interview Questions Guide.

## Data Availability

The datasets generated and/or analysed during the current study are not publicly available due [due to ethics committee requirements and the need for further participants’ consent] but are available from the corresponding author (after getting further ethics approval) on reasonable request.

## Abbreviations

GP: General practitioner
DR: Diabetic retinopathy
MBS: Medicare Benefits Schedule
WA: Western Australia
NSW: New South Wales
CSIRO: Commonwealth Scientific and Industrial Research Organisation
CPD: Continuing Professional Development
NHMRC: National Health and Medical Research Council
NHS: National Health Service
AI: Artificial intelligence
