# Supplementary material for: Barriers and facilitators to diabetic retinopathy screening within Australian primary care
Source: BMC Fam Pract. 2021 Nov 30;22:239. doi: 10.1186/s12875-021-01586-7 (PMC8630186; doi:10.1186/s12875-021-01586-7)
Supplement: Supplementary file 2 — Additional file 2. Interview Questions Guide. [file 12875_2021_1586_MOESM2_ESM.docx]

**Interview Questions Guide**

**Intro**

Tell us beefily about yourself, qualification, clinical experience, practice setting and location.

**GPs’ role in the management of chronic diseases**

- What do you think the role of GPs is in screening or managing chronic diseases and their complications?
- On average, how many patients with diabetes do you see weekly?
- How frequently do you assess diabetic risk factors (HbA1c, lipid profile, proteinuria) of patients?

**Retinal examination in general practice**

- What is the role of GPs in screening for DR in general practice?
- In general, outline your approach to a patient you believe is at risk for DR?
- Do you have a system that sets a schedule/reminder for patients who require retinal screening?
  - What do you have to do if patients don’t attend?
  - Are there suggestions to improve the way your patients are invited about retinal screening, which would improve uptake?
- Are you comfortable with performing visual acuity or fundoscopy (undilated or dilated)?
  - If yes, how frequently will you do fundoscopy in your practice?

**Retinal photography in general practice**

- Do you have a retinal camera in your practice?
  - If so, what type of camera and its cost?
  - Are you happy with the investment?
  - Who uses the camera (nurses or yourself)?
  - How long does it take to obtain a retinal image?
  - How frequently do you retinal photography in practice?
- Do you analyse retinal images by yourself or have image analysis software?
  - If you diagnose DR by yourself, what severity assessment scale you used for DR?
  - What criteria do you use for patients’ referrals?
  - If you use image analysis software, what does it cost?
- If you are uncomfortable with doing DR screening yourself – to whom do you refer patients?
  - If you consult with an ophthalmologist, how long does it take to get a result?
- What kind of follow-up do you once the patient with diabetes has been screened?
- Are you familiar with any guidelines on screening, diagnosis, and management of DR (e. g. NHRMC or RANZGP)?
  - How often will you refer to these guidelines?

**Reimbursement and costs**

- What kind of reimbursement do you get for DR screening?
- Have you heard or used MBS items 12325 or 12326 previously?
  - Were you aware of it beforehand?
  - How did you become aware?
  - What do you think are the pros and cons of these items?
  - Why do you think there has been low uptake of these MBS? Should it be unbundled?
- Are there other costs that aren’t being considered?
- Are you aware of how much a camera cost?
  - How much would you be willing to pay for a camera, given the Medicare rate?
- Do rural practices need subsidies with capital costs?

**Role of technology in DR screening**

- How do you see technology changing DR screening?
- Do you use telehealth in your clinical practice?
  - Can you think of any specific barriers to using technology in DR screening?
- How do you see artificial intelligence screening tools might help in DR screening?
  - What are the issues with this technology?
  - Who takes responsibility for the results using this system?

**Other barriers**

- Why do you think some patients don’t attend the retinal screening appointment?
- Are you able to name barriers to conduct DR screening in your practice?
- What kind of factors do you think might increase the likelihood of introducing DR screening into practice?
